# Selection of indigenous starter culture for safety and its effect on reduction of biogenic amine content in Moo som

**DOI:** 10.5713/ajas.18.0596

**Published:** 2019-02-07

**Authors:** Pussadee Tangwatcharin, Jiraroj Nithisantawakhup, Supaluk Sorapukdee

**Affiliations:** 1 Department of Animal Production Technology and Fisheries, Faculty of Agricultural Technology, King Mongkut’s Institute of Technology Ladkrabang, Bangkok 10520, Thailand

**Keywords:** Lactic Acid Bacteria Starter Culture, *Lactobacillus plantarum*, Antibiotic Resistance, Biogenic Amine, Moo som, Traditional Thai Fermented Pork

## Abstract

**Objective:**

The aims of this study were to select one strain of *Lactobacillus plantarum* (*L. plantarum*) for a potential indigenous safe starter culture with low level antibiotic resistant and low biogenic amine production and evaluate its effect on biogenic amines reduction in Moo som.

**Methods:**

Three strains of indigenous *L. plantarum* starter culture (KL101, KL102, and KL103) were selected based on their safety including antibiotic resistance and decarboxylase activity, and fermentation property as compared with a commercial starter culture (*L. plantarum* TISIR543). Subsequently, the effect of the selected indigenous safe starter culture on biogenic amines formation during Moo som fermentation was studied.

**Results:**

KL102 and TISIR 543 were susceptible to penicillin G, tetracycline, chloramphenicol, erythromycin, gentamycin, streptomycin, vancomycin, ciprofloxacin and trimethoprim (MIC90 ranging from 0.25 to 4 μg/mL). All strains were negative amino acid-decarboxylase for lysis of biogenic amines in screening medium. For fermentation in Moo som broth, a relatively high maximum growth rate of KL102 and TISIR543 resulted in a generation time than in the other strains (p<0.05). These strain counts were constant during the end of fermentation. Similarly, KL102 or TISIR543 addition supported increases of lactic acid bacterial count and total acidity in Moo som fermentation. For biogenic amine reduction, tyramine, putrescine, histamine and spermine contents in Moo som decreased significantly by the addition KL102 during 1 d of fermentation (p<0.05). In final product, histamine, spermine and tryptamine contents in Moo som inoculated with KL102 were lower amount those with TISIR543 (p<0.05).

**Conclusion:**

KL102 was a suitable starter culture to reduce the biogenic amine formation in Moo som.

## INTRODUCTION

Moo som is a traditional Thai fermented pork which is composed of sliced, cut or diced pork, cooked rice, salt and shopped garlic. The mixture is tightly wrapped in banana leaves or packed in plastic bags and then held at room temperature for 2 to 5 d. This product is consumed both without and with cooking and commonly consumed in the north-eastern region of Thailand [1]. The traditional fermented meat products involve indigenous lactic acid bacteria (LAB) that are generally found in raw materials. LAB convert glucose to lactic acid. This increasing lactic acid was observed with decreasing pH which has a preservative effect against competitive microflora during fermentation. In this case, the fermentation is not controlled which affect the organoleptic quality and safety of the final products. Depending on which species of indigenous microbes were present at the start of fermentation, the appearance of pathogenic species such as *Salmonella* spp., *Escherichia coli* and *Staphylococcus aureus* were especially likely to be found in cuisines with pH value more than 4.6 [2]. Some microflora, Enterobacteriaceae, could convert amino acids to biogenic amines by their decarboxylase activities [3]. A trend has emerged which involves the isolation of indigenous strains from traditional fermented meat to be used as potential starters in meat fermentation [4]. These potential starters possess inherent functional characteristics. They can improve food quality and safety (probiotics) by offering one or more characteristics such as antibiotic susceptibility or negative decarboxylase activity [5,6]. Thus, the use of cautiously selected indigenous strains as starters or co-cultures in fermentation processes can help to achieve in situ expression of the required fermentation, retaining a completely natural product and still function as safety starter cultures where applicable.

*Lactobacillus plantarum* (*L. plantarum*) is pervasive lactic acid bacterium and it is detected in environments such as food (fermented meat, dairy products, vegetables, fruits, and beverages), gastrointestinal, respiratory and genital tracts of humans and animals [4]. Various *L. plantarum* isolates have the ability to survive gastric transit and to colonize the intestinal tract of humans and other mammals. Previous study showed that consumption of *L. plantarum* reduced contamination of faecal Enterobacteriaceae and decreased certain risk factors for coronary artery disease. It may result in a dose-dependent reduction in the symptoms of Irritable Bowel Syndrome. The functional food products containing probiotic strains of *L. plantarum* are commercially available [7]. This Lactobacillus is considered as generally regarded as safe in the USA. *L. plantarum* is shown to confer a health benefit on humans and animals [8]. However, the detection of antibiotic resistant strains of *L. plantarum* isolated from fermented meat resulted in their recognition as reservoir of antibiotic resistant genes horizontally transmissible to pathogens through the food chain, this being a matter of concern [9,10].

Generally, relatively high levels of biogenic amines have been found in fermented meat products. Biogenic amines are nitrogenous basic compounds which have objectionable physiological effects on human health. They may be responsible for allergic reactions, nausea, hypotension or hypertension, palpitations, intracerebral haemorrhage and death in very acute cases. In fermented meat products, the main biogenic amines are tyramine, putrescine, histamine, cadaverine, tryptamine, spermine and spermidine. These biogenic amines are commonly products of amino acid-decarboxylase activities of microbes. The most frequent foodborne intoxication is caused by biogenic amines, especially tyramine and histamine [11]. The formation of biogenic amines in fermented sausages is dependent upon many variables including the hygiene of the meat ingredients, method of manufacture, qualitative and quantitative composition of the microflora [12]. Furthermore, the choice of starter cultures is based on inhibiting the increase of the potential aminogenic endogenous bacteria together with their own inability to produce biogenic amines. The five strains of *L. plantarum* demonstrating biogenic amine reduction were apprised. Two strains of *L. plantarum* presented the highest ability to reduce putrescine and tyramine in culture media [13]. Starter culture *L. plantarum* was efficient in reducing cadaverine, putrescine, histamine, tryptamine, phenylethylamine, and tyramine accumulation in som-fug [14]. A strains of *L. plantarum* isolated from fermented sausages had a strong effect on inhibiting the biogenic amines production in sausage fermentation. The selection of suitable inoculating starter culture has been one of the most effective strategies to prevent or minimize the presence of biogenic amines [15].

Our previous studies reported that three strains of *L. plantarum*, KL101, KL102, and KL103 were isolated from traditional Thai fermented meat for indigenous starter culture strains. These bacteria exhibited antimicrobial activity against *Staphylococcus aureus*, *Escherichia coli*, *S.* Typhimurium and *L. monocytogenes* [16] and survival in the gastrointestinal environment (acidity, pepsin, bile salt, and pancreatin) [17] and were selected candidate probiotics. In this study, the selection of indigenous safe strain intended as a Moo som starter culture and its influence on biogenic amine accumulation in Moo som was investigated.

## MATERIALS AND METHODS

### Strains and starter culture preparation

Three strains of *L. plantarum*, *L. plantarum* KL101 (KL101), *L. plantarum* KL102 (KL102), and *L. plantarum* KL103 (KL103) were previously isolated from traditional Thai fermented meat as indigenous starter cultures. Briefly, these strains were selected for their preliminary probiotic function, including antimicrobial activity against foodborne pathogens [16] and survival in the gastrointestinal environment [17]. *L. plantarum* TISIR543 (TISIR543) as commercial starter culture was purchased from Culture Collection of National Center for Genetic Engineering and Biotechnology (BIOTIC), Thailand. Each strain was stored at −20°C in de Man, Rogosa and Sharpe (MRS) broth (Merck, Darmstadt, Germany) containing 20% (v/v) glycerol. Frozen cultures were cross-linked on MRS agar and then incubated anaerobically at 30°C for 24 h prior to use. Cells were harvested by centrifugation at 6,000×g at 4°C for 10 min in Universal 16R refrigerated centrifuge (Hettich Zentrifugen, Tuttlingen, Germany), washed and resuspended in saline solution (0.85% (w/v) NaCl). Finally, the cell concentration of each LAB starter culture was adjusted to 5×10^8^ cfu/mL with saline solution and a McFarland standard turbidity of 0.5.

### Screening of safety lactic acid bacteria starter cultures

#### Antimicrobial susceptibility testing

The antimicrobial resistance was estimated using the broth microdilution method which was modified from Dušková and Karpíšková [18]. The following antibiotics were tested in MRS broth: 0.0625 to 256.0000 μg/mL of penicillin G, tetracycline, chloramphenicol, erythromycin, gentamycin, streptomycin, vancomycin, ciprofloxacin and trimethoprim. Each LAB suspension was inoculated in microtiter plate at final concentration of 5×10^5^ cfu/mL and then incubated anaerobically at 30°C for 24 h. The minimum inhibitory concentrations (MIC) were determined. The strains were divided as susceptible (MICs <8 μg/mL), moderately resistant (MICs 8 to 32 μg/mL) or resistant (MICs >32 μg/mL) base on the MIC requirement that the antibiotic inhibit the growth of 90% of bacteria (MIC90) as indicated by Walsh [19].

#### Testing for the decarboxylase activity of lactic acid bacteria starter cultures

In order to support the enzyme induction before the actual screening test, LAB strains were subcultured 3 times in MRS broth, containing 0.1% (w/v) of each precursor amino acid (Merck, Germany), including L-histidine monohydrochloride, L-ornithine monohydrochloride, L-lysine monohydrochloride, L-arginine monohydrochloride and tyrosine disodium in addition to supplementation with 0.005% (w/v) of pyridoxal-5-phosphate hydrate and 0.01% (w/v) thiamine hydrochloride (Merck, Germany) and then incubated anaerobically at 30°C for 24 h. All strains were streaked on MRS agar, containing 0.1% (w/v) of each precursor amino acid, 0.005% (w/v) of pyridoxal-5-phosphate hydrate and 0.01% (w/v) thiamine hydrochloride and then incubated anaerobically at 30°C for 24 h [20].

All strains of LAB were assayed for decarboxylase activities in screening medium. The decarboxylase activities were tested by inoculating individual LAB colonies from MRS agar, containing 0.1% (w/v) of each precursor amino acid, directly onto plates containing the modified decarboxylase medium, including 0.005% (w/v) of pyridoxal-5-phosphate hydrate, modified from method of Maijala [21]. The medium included the precursor amino acids at 1% (w/v) final concentration. Purple bromocresol was contained as pH indicator. The pH was adjusted to 5.3 and then the medium was autoclaved. The screening medium without amino acid was used as control. The inoculated plates were incubated anaerobically at 30°C for seven days. The conversion of clear zone around colony, color changing from purple to yellow, was determined as amino acid decarboxylase positive. Therefore, another result was determined as amino acid decarboxylase negative.

### Fermentation of *Lactobacillus plantarum* in Moo som broth

Moo som broth was prepared by using a method modified from Swetwiwathana et al [22]. Moo som broth included 0.90% (w/v) meat extract, 0.90% (w/v) tryptone, 0.05% (w/v) sodium ascorbate, 0.3% (w/v) sodium tripolyphosphate, 0.90% (w/v) glucose, 2.40% (w/v) sodium chloride (all reagents from Merck, Germany), 0.20% (w/v) cooked rice. The pH was adjusted to 6.8 and then the medium was autoclaved. The sterilized garlic of 5% (w/v) and sodium nitrite of 0.008% (w/v) were added in sterilized medium which garlic was sterilized by soaking in 70% (v/v) ethanol solution for 20 min. Moo som broth was stored 2°C until using. Four separated media of Moo som broth were inoculated with different strains, TISIR543, KL101, KL102, and KL103 at an initial concentration at 10^5^ cfu/mL. All samples were incubated anaerobically at 30°C for 72 h and then taken at 0, 3, 6, 9, 12, 15, 18, 21, and 24 h for analysis of LAB growth, pH and total acidity.

#### Microbiological analysis

LAB growth was performed in two replicates during the fermentation. One milliliter of sample was diluted in 9 mL of saline solution. The diluted solution was serially diluted in saline solution and 0.1 mL of each dilution was grown on MRS agar and then incubated anaerobically at 30°C for 24 to 48 h [23]. After that, the maximum growth rate (μ_max_) and generation time (λ) were calculated according to Oliveira et al [24] as following equations:

(1) $$\mu_{\text{max}}=\text{ln}({\text{X}}_{2}/{\text{X}}_{1})/({\text{t}}_{2}-{\text{t}}_{1})$$

Where: X_1_ and X_2_ are LAB counts; t_1_ and t_2_ are incubation times.

(2) $$\lambda =\text{ln }2/\mu_{\text{max}}$$

#### Determination of pH and total acidity

Twenty-five milliliters of sample was taken for pH and total acid estimation. Both estimations were estimated triplicates for each supernatant fluid. Direct pH estimation was taken by using pH meter (Mettler Toledo S20, Schwerzenbach, Switzerland). The amount of the total acid as lactic acid produced in the fermentation of Moo som broth was estimated by the standard titration procedure for total titratable acidity according to AOAC [25]. Total acid content estimation was done by titrating the supernatant fluid of the substrates on addition of phenolphthalein as an indicator, 0.1 M NaOH was titrated into the samples. The total acidity was calculated as lactic acid and expressed as g/100 mL.

### Effects of commercial starter culture and indigenous strain on the microbial changes and biogenic amine contents during fermentation

#### Moo som preparation

The Moo som mixture, common recipe, was prepared with lean pork and the ingredients and additives (g/kg): cooked rice, 65; minced garlic, 65; sugar, 4; erythrobate, 4; sodium tripolyphosphate, 3; NaCl, 10; and potassium nitrite, 0.8. Lean pork was stored at 2°C, sliced and mixed with the other ingredients and additives. Three separated batches of Moo som were prepared with different inoculums (non-inoculum [control], TISIR543, and KL102) at initial concentration of 10^5^ cfu/g. Each mixture batch was stuffed into plastic casing with a diameter 30 mm (approximately 100 g each) and sealed tightly. All samples were incubated at 30°C for 3 d. They were taken at every 1 d for analysis of microbiology, pH and total acidity and taken at 0, 1, 2, 3, 5, and 7 d for analysis of biogenic amines.

#### Microbiological analysis

The microbiological analyses were performed in two replicates during the fermentation for LAB, Staphylococci and Enterobacteriaceae. The casings were aseptically removed and 25 g of Moo som sample was diluted in 225 mL of saline solution and homogenised in a stomacher bag mixer (Interscience, Saint Nom la Bretèche, France). The homogenate was serially diluted with saline solution and each dilution was grown in different growth media. The following media and incubated conditions were used: i) MRS agar and then incubated anaerobically at 30°C for 24 to 48 h for LAB count [20], ii) mannitol salt agar (Oxoid, Hampshire, UK) incubated at 30°C for 24 to 48 h for Staphylococci [4], and iii) crystal-violet neutral-red bile dextrose agar (Merck, Germany) incubated at 37°C for 24 h for Enterobacteriaceae count [5].

#### Determination of pH and total acidity

The sample homogenates for pH and total acidity determination were prepared. Two grams of sample were added with 20 mL of distilled water and then homogenised with Ultra-Turrax IKA (WERKE GMBH & CO.KG, Staufen, Germany) for 60 s. Triplicate determination for each sample was carried out. Direct pH measurement was taken with a pH meter. The homogenate was centrifuged at 3,000×g for 15 min. The supernatant was filtered through Whatman filter paper No. 4 (Merck, Darmstadt, Germany). The filtrate was titrated with 0.1 M NaOH using phenolphthalein as an indicator. The total acidity was calculated as lactic acid and exposed as g/100 g [26].

### Determination of biogenic amine

Meat and Moo som samples were prepared for determination of biogenic amines by using a method modified from Tosukhowong et al [5]. Samples were cut into small pieces and blended with a blender (Ronic, Vitry en Charollais, France) for 30 s at 2 times. Five grams of blended sample was taken into plastic bag and extracted with 0.3 M tricholoacetic acid (Sigma Chemical, St. Louis, MO, USA) using the stomacher bag mixer for 8 min. A 500 μL of 77 mM 1, 7-diaminoheptane (Sigma Chemical, USA) was using as an internal standard. The ratio of sample and 0.3 M tricholoacetic acid was 1:5 (w/v). The supernatant was collected by centrifugation at 4,000×g at 4°C for 15 min and then kept at −20°C for high performance liquid chromatography derivatization.

The method for determining biogenic amines was modified from Tosukhowong et al [5]. Freshly prepared dansyl chloride (37 mM in acetone) was used as a derivatizing agent. A 300 μL of supernatant or standard solution was mixed with 60 μL of 2 M NaOH and 90 μL of a saturated solution of sodium hydrogen carbonate. The reaction mixture was leaved at room temperature for 30 min. To this was added 600 μL of fresh dansyl chloride solution and incubated at 40°C for 45 min. The reaction mixture was stopped by 30 μL of 32% ammonia solution (Merck, Germany) and then left at room temperature for 30 min. The sample volume was adjusted with acetonitrile (Sigma Chemical, USA) to 1,500 μL of final volume and gently mixed. Sample was centrifuged at 3,000×g at 4°C for 5 min. The supernatant was filtered through a Minisart RC4 filter (0.45 μm pore size, Sartorius, Goettingen, Germany). After that, 20 μL of filtrate was injected into HPLC for analysis.

Biogenic amine was separated on Luna NH2 column, 4.6× 250 mm, 3 μm (Phenomenex, Torrance, CA, USA) and the analyte quantified on Thermo separation products model ConstaMetric 4100 Bio (Mundelein, IL, USA). The temperature of column was set at 40°C. A 20 μL of derivatized sample or standard was injected. The mobile phase was composed of 0.1 M ammonium acetate (Sigma Chemical, USA) as solvent A and acetonitrile as solvent B. The flow rate was 1.2 mL/min. The isocratic program started at 10% A and 10% B within 10 min and held for 5 min before starting the next run. After that, solvent B was raised to 90% within 25 min. Biogenic amines were detected at wavelength 254 nm by a UV detector (Shimadzu, Kyoto, Japan). The biogenic amine concentration in pork and Moo som was calculated by comparing with the standard concentration.

### Statistical analysis

Data are shown as means and standard deviations. Results were based on analysis of the general linear model by SAS 9.0 software (SAS Institute, Cary, NC, USA), except MIC and MIC90 were assessed by the division of antimicrobial agent in three classes [19]. Pearson’s correlation coefficients were carried out to determine the relationship among variables using the CORR procedure. A selected indigenous strain of *L. plantarum* was examined by MICs of all antibiotics as the susceptible class, negative amino acid-decarboxylase in screening medium, the highest maximum growth rate and the lowest generation time in Moo som broth. After that, this selected strain was studied its effect on formation of biogenic amines in Moo som.

## RESULTS AND DISCUSSION

### Screening of safety lactic acid bacteria starter cultures

The present study determined the lowest antibiotic concentration that inhibit (MIC) and 90% (MIC90) of the tested LAB strains. The results showed that TISIR543 and KL102 were observed to be susceptible to all antibiotics while KL101 and KL103 were observed to moderately resistant to tetracycline, gentamycin and streptomycin, ciprofloxacin and trimethoprim (Table 1). Generally, most lactobacilli are intrinsically resistant to aminoglycosides (streptomycin and gentamycin), glycopeptides (vancomycin), inhibitors of nucleic acid synthesis (ciprofloxacin) and inhibitors of folic acid synthesis (trimethoprim). However, they are susceptible to penicillins, chloramphenicol, streptomycin, tetracycline and erythromycin [27–29]. Additionally, all *L. plantarum* strains were negative amino acid-decarboxylase for lysis of L-histidine monohydrochloride, L-ornithine monohydrochloride, L-lysine monohydrochloride, L-arginine monohydrochloride, and tyrosine disodium in the screening medium. For a safety starter culture, the antibiotic resistant results showed that TISIT543 and KL102 was safer than KL101 and KL103.

### Effect of different strain of starter culture during fermentation of Moo som broth

**Figure 1:**
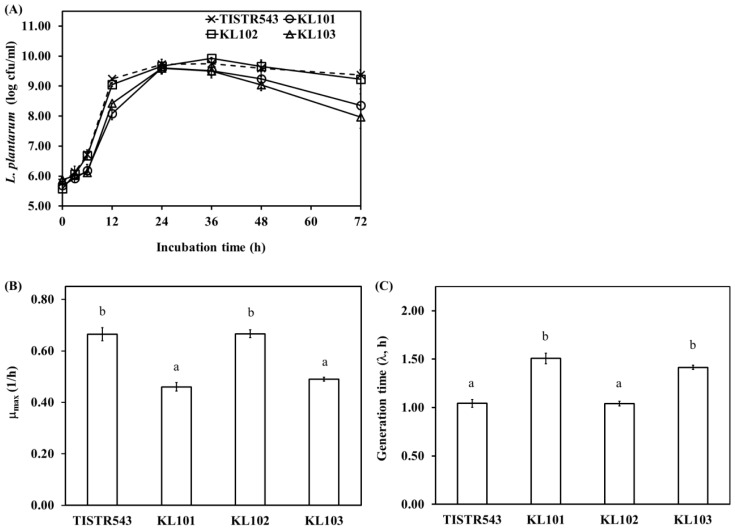
*Lactobacillus plantarum* counts (A), maximum growth rate (μ_max_) (B) and generation time (λ) (C) of *Lactobacillus plantarum* in Moo som broth during fermentation. Different lowercase letters indicate significant differences in sample (p<0.05).

**Figure 2:**
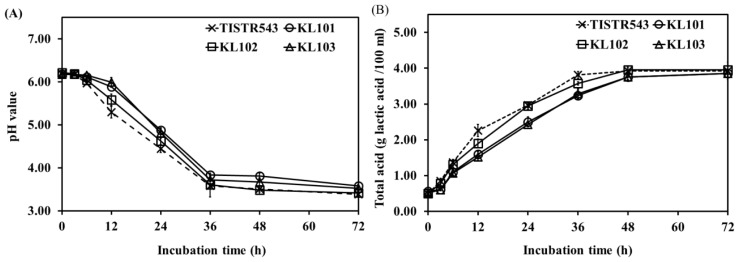
pH values (A) and total acid (B) in Moo som broth during fermentation.

*L. plantarum* count indicated significant differences between Moo som broth inoculated with different strains (p<0.05) during fermentation. At the beginning of fermentation, TISIR543 and KL102 counts were higher than KL101 and KL103 after 12 h of fermentation (p<0.05) (Figure 1A). Due to relatively high maximum growth rate of TISIR543 and KL102 (0.66±0.03 and 0.67±0.02 L/h, respectively) (Figure 1B), generation times of TISIR543 and KL102 were lower than those of KL101 and KL103 (p<0.05) (Figure 1C). However, all strains grew up to 24 h of fermentation (<10^10^ cfu/g). Thereafter, TISIR543 and KL102 counts were constant throughout until the end of fermentation, while KL101 and KL103 counts decreased moderately over the same fermentation time. The decreased counts of KL101 and KL103 in Moo som broth resulted from low pH values in Moo som broth (pH 3.42 to 3.84) after 36 to 72 h of fermentation (Figure 2A), which was non-optimal pH for *L. plantarum* growth (pH<4) [30]. Although there were differences in growth rate between strains, the cultures generally exhibited an increase sensitivity at pH values below 4.0. Acid tolerance was accepted as one of the desirable properties used to select potentially probiotic strains [31]. Hence, for growth and count consistence of starter culture, KL102 was a potential indigenous starter culture and selected to be applied in Moo som production as safety starter culture.

The results of pH and total acid content in Moo som broth inoculated with TISIR543, KL101, KL102, and KL103 were observed as the fermentation progressed (Figure 2). Within the 36 h of fermentation, the pH value was significantly decreased in Moo som broth inoculated with all strains (p<0.05). After that, no differences in pH values were observed between fermentation times until the end of fermentation (after 72 h of fermentation) (p>0.05). Similarly, total acid content in all samples increased rapidly after 48 h of fermentation. Then, total acid contents were constant throughout the remaining fermentation time. Commonly, homo- or heterofermentative LAB produce lactic acid as their major end-product of fermentation via the Embden-Meyerhof pathway (glycolysis) or the phosphoketolase pathway (phosphogluconate pathway), respectively [32]. The sharp decline of pH in early fermentation was accompanied with an increase in *L. plantarum* counts and total acid (r = −0.819, p<0.01 and r = −0.982, p<0.01, respectively). In addition, a fast growth of LAB leading to rapid pH decline within 36 h is necessary to guard against growth of spoilage and pathogenic bacteria [33].

### Microbiological changes in Moo som inoculated with commercial starter culture and indigenous strain during fermentation

**Figure 3:**
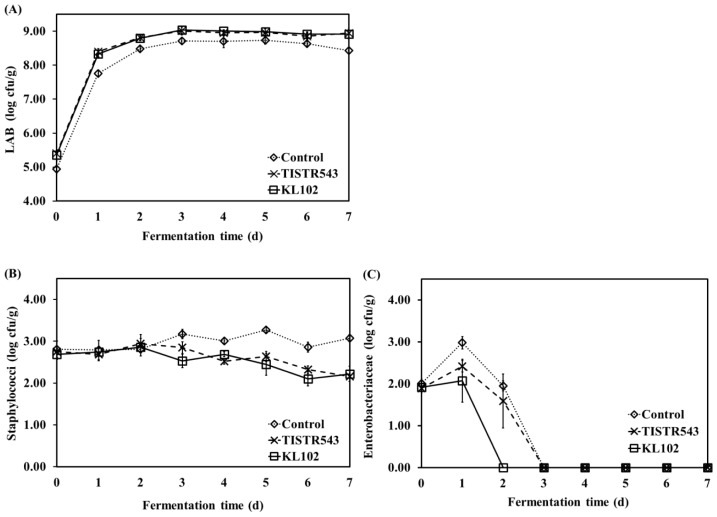
Lactic acid bacteria (LAB) (A), Staphylococci (B) and Enterobacteriaceae (C) counts (log cfu/g) of Moo som during fermentation.

LAB, Staphylococci and Enterbacteriaceae counts during fermentation of Moo som without and with inoculation of *L. plantarum* starter culture are summarized in Figure 3. The initial microflora of all samples was obtained from the raw minced pork, except inoculated starter culture samples which TISIR543 and KL102 were inoculated at final concentration of 10^5^ cfu/g. For raw minced pork, counts of LAB, Staphylococci and Enterbacteriaceae were 4.63±0.17, 2.78±0.24, and 2.06±0.09 log cfu/g, respectively. During fermentation of all samples, LAB increased rapidly and reached to a maximum 10^8^ cfu/g after 3 d of fermentation and then these counts were constant untill the end of fermentation. However, LAB count in inoculated samples was higher than control sample during fermentation (p<0.05) (Figure 3A). Figure 3B shows that Staphylococci count of inoculated samples decreased slightly at the end of fermentation while this count in the control sample was constant during fermentation. According to previous study, Staphyloccoci counts of Nham without the addition of inoculum remained constant throughout the fermentation period, possibly due to the decrease in pH [5]. On the other hand, Enterobacteriaceae count increased after 1 d of fermentation and then decreased rapidly in all samples, which was lower than 1 log cfu/g after 2 d of fermentation for Moo som inoculated with KL102 and after 3 d of fermentation for Moo som inoculated with TISIR543 and control (Figure 3C), possibly due to decrease in pH value. The rapid decrease of Enterobacteriaceae count during fermentation was accompanied with an increase in total acid and a decrease in pH (r = −0.862, p<0.01 and r = 0.801, p<0.05, respectively). In Moo som, a rapid growth of LAB caused a total acid increase and pH to decrease to below 4.6 within 3 d, which is essential to inhibit growth of unfavorable microflora [30]. Additionally, various metabolic products of LAB, such as short-chain organic acids, hydrogen peroxide, carbon dioxide, diacetyl and bacteriocin have antimicrobial potential [34].

### pH and total acidity measurement

**Figure 4:**
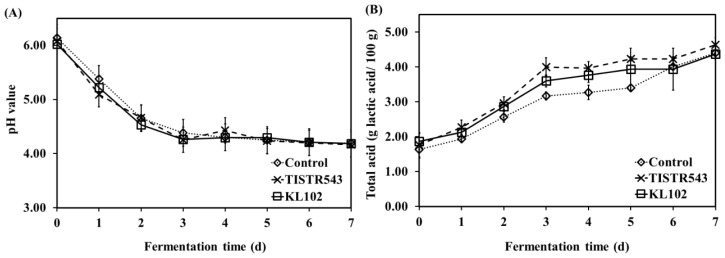
pH values (A) and total acid content (B) of Moo som during fermentation.

The pH and total acid content results are shown in Figure 4. All Moo som samples presented sharp decrease in pH during the first 2 d of fermentation and continuously decreased to a final pH value of lower than 4.6 after 3 d of fermentation (Figure 4A). Similarly, as fermentation time increased, total acid contents of all samples increased quickly during the first 3 d of fermentation and then remained nearly constant throughout 7 d of fermentation (Figure 4B). The sharp decrease in pH value during fermentation was accompanied with an increase in total acidity (r = −0.897, p<0.01).

### Effects of commercial starter culture and indigenous strain on the biogenic amine contents during fermentation

In fresh minced pork, the physiological polyamines (spermine and spermidine) and diamines (histamine and putrescine) were found as the main amines with values of 98.20±1.49, 51.81±2.53, 73.21±1.48, and 71.10±5.40 mg/kg, respectively. However, the monoamine tyramine and diamine tryptamine were found in lower amounts with values of 24.40±2.91 and 23.89±0.10 mg/kg. However, cadaverine was not detectable. According to the previous study, the most common amine was spermine, while cadaverine was not found in fresh pork [5].

Biogenic amine accumulation during Moo som fermentation inoculated with commercial starter, TISIR543, or indigenous starter culture, KL102 was observed at 0, 1, 2, 3, 5, and 7 d and compared with that of naturally fermented Moo som, control (Table 2). Overall, tyramine, putrescine, histamine and spermidine were influenced by different starter cultures in Moo som fermentation. Tyramine formation started after 1 and 2 d of fermentation in Moo som for without and with starter culture inoculations, respectively. After 1 and 5 d of fermentation tyramine contents were significantly higher in control sample and sample inoculated with TISIR543, respectively, than in sample inoculated with KL102 at the same fermentation time. Tyramine is the biogenic amine most generally related to various species LAB found in fermented sausage which biosynthesizes tyramine [5,35]. In control Moo som, a rapid increase of tyramine formation during fermentation was accompanied with an increase in LAB loading (r = 0.859, p<0.05). The tyramine toxic level is 100 to 800 mg/kg [36]. In this research, tyramine contents in naturally inoculated Moo som and Moo som inoculated with commercial starter culture after more than 2 and 7 d of fermentation (over fermentation), respectively, were higher than 100 mg/kg. In contrast, in Moo som inoculated with KL102 the tyramine content was lower than 100 mg/kg throughout fermentation, therefore this represents a safe level. The formation of putrescine depended on the inoculum strain. Moo som inoculated with KL102 accumulated constant amounts of putrescine throughout fermentation due to relative decrease in Enterobacteriaceae count during early fermentation. The appearance of putrescine in meat products has been associated with the ornithine-decarboxylase activity of Enterobacteriaceae [3,35]. However, histamine content was lower in Moo som inoculated with KL102 throughout fermentation (p<0.05). The histamine accumulation in all samples was lower than 100 mg/kg which should be the upper limit for potential risk to healthy individuals [36]. Furthermore, spermine accumulation in Moo som inoculated with KL102 varied slightly during fermentation, while this amine in naturally inoculated Moo som and Moo som inoculated with TISIR543 increased rapidly after 2 and 5 d of fermentation, respectively. A previous study reported that amine oxidase of some indigenous strains of *L. plantarum* were able to degrade spermine [13]. On the other hand, spermidine content in all samples decreased during early fermentation and was not found after 3 d of fermentation since spermidine can be consumed as a nitrogen source by the microorganisms [37]. Moreover, levels of tryptamine varied slightly during fermentation while its level in all samples was lower than 50 mg/kg. Special concern is devoted to a group of polyamines; putrescine, spermidine and spermine. In humans, polyamines are taken up and used by the fast dividing tissues, consequently polyamines are concentrated in tumors [38]. However, cadaverine was not detectable in all Moo som fermentations. Therefore, biogenic amine formation in Moo som could be prevented by the addition of KL102. Due to the limited knowledge about biogenic amine reduction, the mechanism by which KL102 can degrade some biogenic amines content in Moo som should be further studied.

Results of this study indicate that *L. plantarum* KL102 is a candidate indigenous starter culture probiotic with low level antibiotic resistance and low decarboxylase activities. Furthermore, this strain showed the fastest fermentation rate as a potential indigenous starter culture. For Moo som fermentation, the reduction of tyramine, putrescine, histamine and spermine content was successfully obtained in Moo som inoculated with KL102. Therefore, the addition of KL102 as indigenous starter culture could minimize the biogenic amine formation in Moo som fermentation.

## Figures and Tables

**Table 1:** MIC values for the *Lactobacillus plantarum* investigated given by strains

Antibiotics	*L. plantarum strains*	MIC (μg/mL)	MIC90 (μg/mL)	Interpretation1)
Penicillin G	TISIR543	0.25	0.5	Susceptible
	KL101	0.125	0.25	Susceptible
	KL102	0.25	0.25	Susceptible
	KL103	0.5	0.5	Susceptible
Tetracycline	TISIR543	4	4	Susceptible moderately
	KL101	4	16	Resistant
	KL102	4	4	Susceptible moderately
	KL103	4	8	Resistant
Chloramphenicol	TISIR543	2	4	Susceptible
	KL101	2	4	Susceptible
	KL102	2	4	Susceptible
	KL103	2	4	Susceptible
Erythromycin	TISIR543	2	4	Susceptible
	KL101	1	1	Susceptible
	KL102	1	4	Susceptible
	KL103	1	2	Susceptible
Gentamycin	TISIR543	2	4	Susceptible
	KL101	4	8	Moderately resistant
	KL102	2	4	Susceptible
	KL103	4	16	Moderately resistant
Streptomycin	TISIR543	2	4	Susceptible moderately
	KL101	8	16	Resistant
	KL102	4	4	Susceptible
	KL103	16	16	Moderately resistant
Vancomycin	TISIR543	0.5	1	Susceptible
	KL101	0.25	1	Susceptible
	KL102	0.25	0.5	Susceptible
	KL103	0.5	2	Susceptible
Ciprofloxacin	TISIR543	1	2	Susceptible moderately
	KL101	4	8	Resistant
	KL102	2	4	Susceptible
	KL103	16	16	Moderately resistant
Trimethoprim	TISIR543	2	4	Susceptible moderately
	KL101	4	16	Resistant
	KL102	2	2	Susceptible
	KL103	4	8	Moderately resistant

Values are given as means from triplicate determinations.

MIC, minimum inhibitory concentration; *L. plantarum*, *Lactobacillus plantarum*.

1) Subdivision of the antibiotic in three classes in function of their effect on the strains of *L. plantarum* evaluation by their MICs values: susceptible (MICs<8 μg/mL), moderately resistant (MICs 8–32 μg/mL) or resistant (MICs>32 μg/mL) [16].

**Table 2:** Biogenic amine accumulation in Moo som during fermentation

Biogenic amine	Samples	Fermentation time (d)					

		0	1	2	3	5	7
Tyramine	Control	21.74±4.74^a^^A^	61.43±9.65^b^^B^	122.90±2.49^b^^D^	171.12±11.27^b^^E^	92.66±6.34^c^^C^	165.81±4.80^c^^E^
	TISTR543	36.79±0.25^b^^A^	50.03±4.28^a^^B^	72.34±6.84^a^^B^	67.13±4.71^a^^B^	74.56±5.66^b^^B^	132.70±9.43^b^^C^
	KL102	38.77±1.37^b^^A^	44.68±3.48^a^^A^	73.57±6.21^a^^C^	58.03±1.34^a^^B^	61.01±3.97^a^^B^	71.06±4.11^a^^C^
Putrescine	Control	87.71±2.53^a^^A^	134.49±9.43^b^^C^	120.84±7.49^a^^BC^	109.60±8.37^b^^B^	111.17±11.08^b^^B^	111.17±6.43^b^^B^
	TISTR543	86.27±8.19^a^^A^	116.52±7.49^b^^B^	107.31±3.85^a^^BC^	95.55±1.39^ab^^A^	109.37±6.94^b^^C^	75.34±2.80^a^^A^
	KL102	85.49±2.99^a^^B^	91.25±7.83^a^^B^	106.25±5.29^a^^C^	86.66±3.71^a^^B^	82.77±6.28^a^^B^	67.15±2.47^a^^A^
Histamine	Control	84.29±6.94^a^^B^	89.12±6.22^c^^BC^	67.65±2.78^b^^A^	65.47±2.94^b^^A^	94.71±3.39^b^^C^	75.66±4.26^b^^B^
	TISTR543	77.34±6.39^a^^B^	60.27±2.64^b^^A^	76.49±3.81^c^^B^	80.06±4.38^c^^B^	89.82±2.72^b^^C^	97.50±1.88^c^^C^
	KL102	85.59±4.39^a^^C^	32.50±2.78^a^^A^	60.64±1.90^a^^B^	55.98±1.58^a^^B^	80.87±3.11^a^^C^	62.16±1.59^a^^B^
Spermine	Control	118.44±4.20^a^^A^	120.77±5.74^c^^A^	154.76±8.99^c^^B^	189.21±8.12^c^^C^	273.04±12.03^b^^D^	259.69±9.24^b^^D^
	TISTR543	112.65±6.84^a^^B^	106.00±2.59^b^^A^	86.56±4.43^b^^A^	115.13±5.38^b^^B^	251.34±8.93^b^^C^	259.69±4.97^b^^C^
	KL102	103.40±8.29^a^^D^	78.94±3.72^a^^C^	58.43±2.45^a^^B^	44.79±2.68^a^^A^	101.95±6.90^a^^D^	83.62±1.48^a^^C^
Spermidine	Control	44.81±1.41^a^^B^	71.47±5.27^b^^C^	4.54±0.89^A^	ND1)	ND	ND
	TISTR543	38.24±3.65^a^^A^	65.11±6.32^b^^B^	ND	ND	ND	ND
	KL102	37.51±1.01^a^^B^	25.09±2.21^a^^A^	ND	ND	ND	ND
Tryptamine	Control	25.55±0.28^b^^B^	14.46±0.18^a^^A^	22.07±2.83^a^^B^	31.40±1.84^b^^C^	32.93±0.31^b^^C^	32.06±4.38^b^^C^
	TISTR543	25.32±0.29^b^^A^	23.49±0.16^c^^A^	37.96±3.48^b^^BC^	39.16±0.57^c^^C^	35.34±4.01^b^^BC^	32.03±0.18^b^^B^
	KL102	16.73±0.14^a^^A^	16.73±0.25^b^^A^	20.21±0.36^a^^AB^	22.28±2.66^a^^BC^	25.43±3.81^a^^C^	16.88±0.43^a^^A^

Values are given as means±standard deviation from triplicate determinations.

ND, not detected.

a–c Different lowercase letters within the same fermentation time indicate significant differences among sample (p<0.05).

A–E Different uppercase letters within the same sample indicate significant differences among fermentation time (p<0.05).

1) Amounts below the detection level.
